# Supplementary material for: Capsular specificity in temperate phages of Klebsiella pneumoniae is driven by diverse receptor-binding enzymes
Source: PLoS Biol. 2026 Apr 28;24(4):e3003716. doi: 10.1371/journal.pbio.3003716 (PMC13123978; doi:10.1371/journal.pbio.3003716)
Supplement: S1 Fig — Each point shows the completeness of each prophage detected in an isolate with the corresponding K-type. Box-plots are overlaid to show the median values and quantiles. (Bottom) The same but shown for a single representative per phage variant with the highest value of completeness. The data underlying this Figure can be found at Figshare (https://doi.org/10.6084/m9.figshare.29181188), and can be reproduced using code archived in Zenodo (https://doi.org/10.5281/zenodo.18699826). (PDF) [file pbio.3003716.s001.pdf]

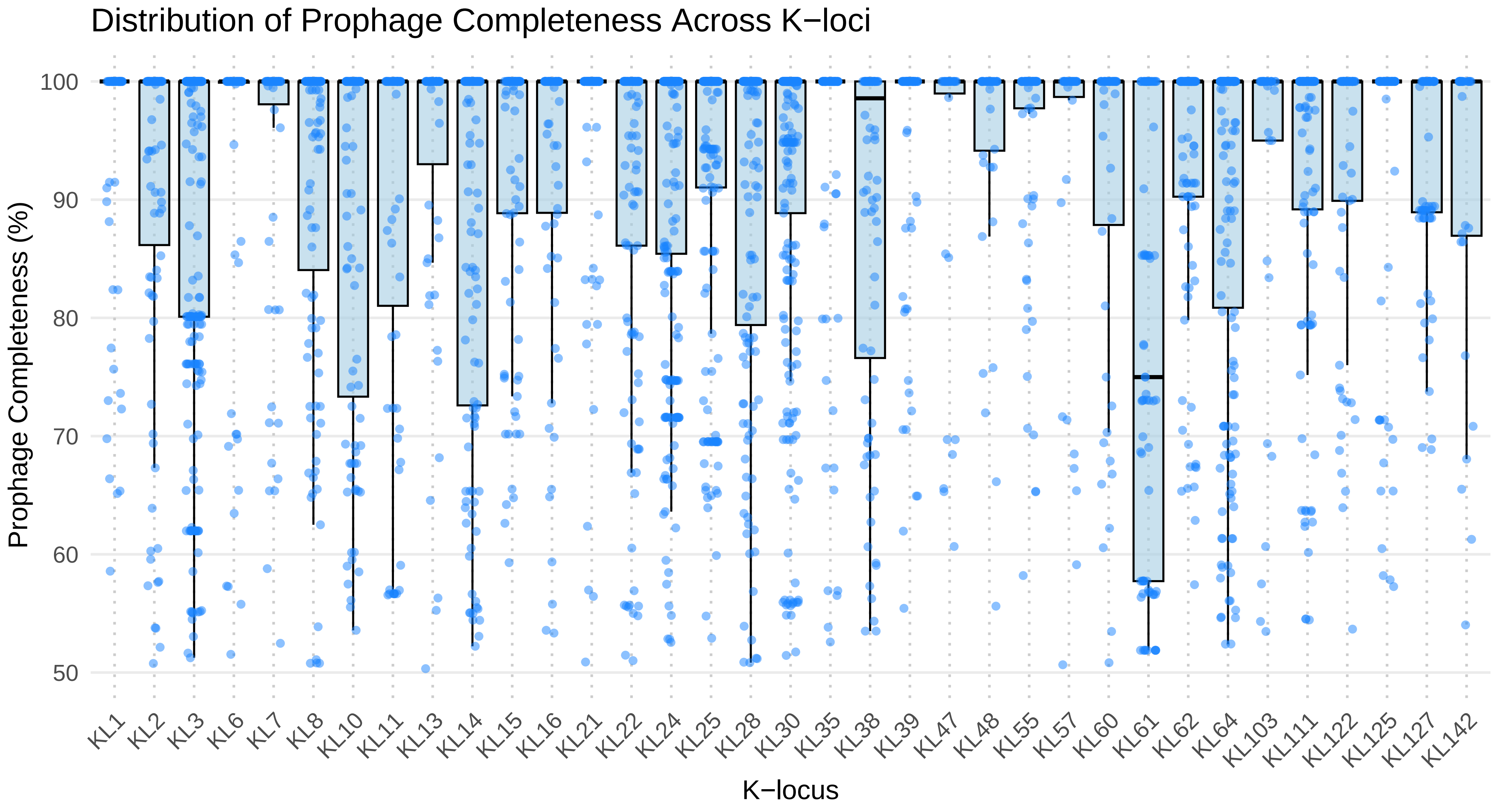

Distribution of Prophage Completeness Across K−loci
Prophage Completeness (%)
100
90
80
70
60
50
KL1 KL2 KL3 KL6 KL7 KL8 KL10 KL11 KL13 KL14 KL15 KL16 KL21 KL22 KL24 KL25 KL28 KL30 KL35 KL38 KL39 KL47 KL48 KL55 KL57 KL60 KL61 KL62 KL64 KL103 KL111 KL122 KL125 KL127 KL142
K−locus

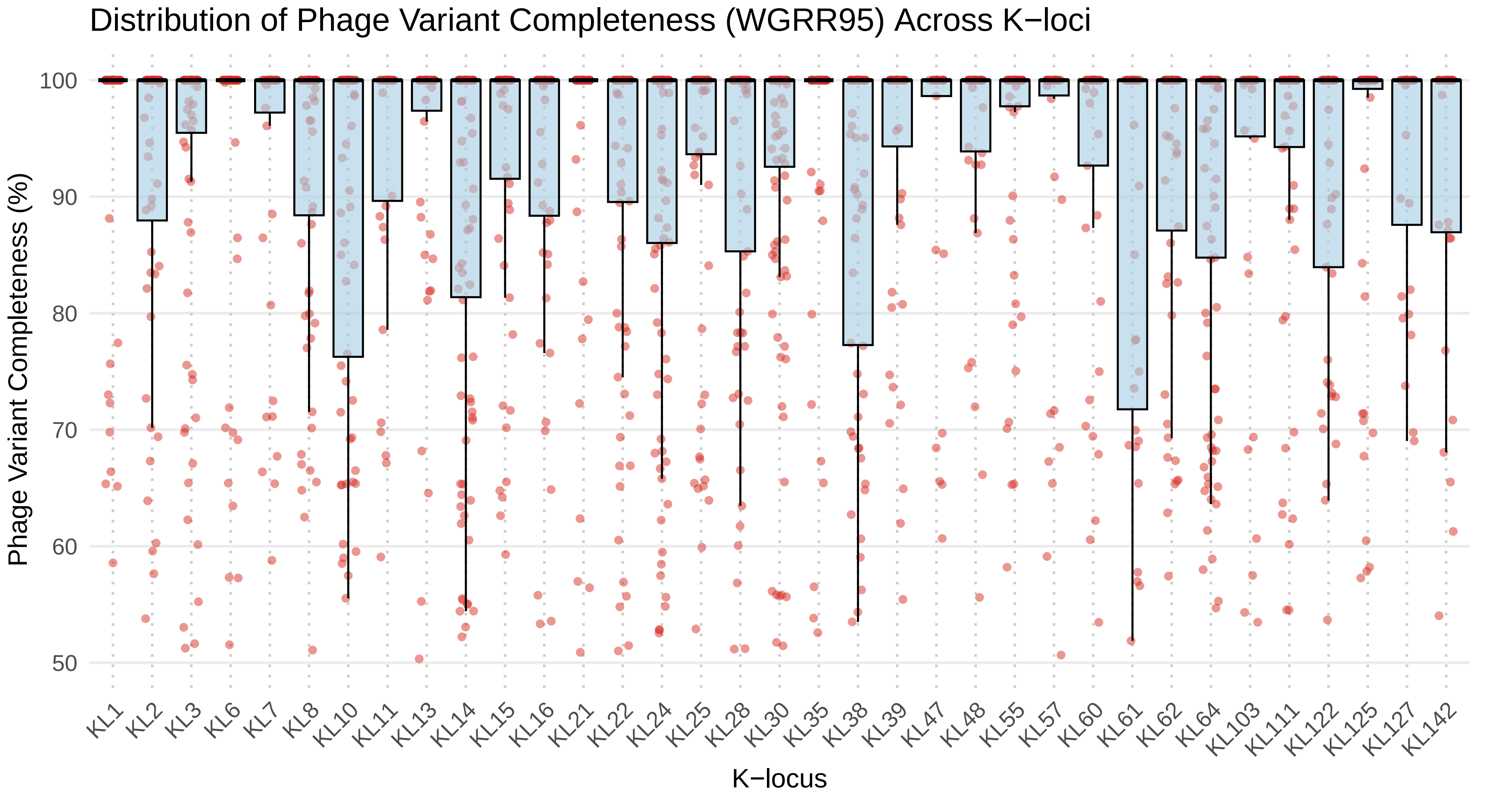

Distribution of Phage Variant Completeness (WGRR95) Across K−loci
Phage Variant Completeness (%)
100
90
80
70
60
50
KL1 KL2 KL3 KL6 KL7 KL8 KL10 KL11 KL13 KL14 KL15 KL16 KL21 KL22 KL24 KL25 KL28 KL30 KL35 KL38 KL39 KL47 KL48 KL55 KL57 KL60 KL61 KL62 KL64 KL103 KL111 KL122 KL125 KL127 KL142
K−locus
