# Supplementary material for: Capsular specificity in temperate phages of Klebsiella pneumoniae is driven by diverse receptor-binding enzymes
Source: PLoS Biol. 2026 Apr 28;24(4):e3003716. doi: 10.1371/journal.pbio.3003716 (PMC13123978; doi:10.1371/journal.pbio.3003716)
Supplement: S2 Text — (PDF) [file pbio.3003716.s022.pdf]

Supplementary Text S2

Experimental validation of capsule depolymerase activity.

Table 1 Experimental results for all active recombinant proteins identified through manual screening. For each protein, the predicted host K-locus (based on prophage origin), experimentally confirmed K-locus specificity, expression level, and minimal halo-forming concentration (MHFC) are shown. Spot tests were performed using both purified proteins (tested at the initial concentration and in a 2-fold dilution series) and unpurified lysates and insoluble fractions to assess activity comprehensively. The lowest concentration at which visible capsule degradation (halo formation) occurred was recorded as MHFC.

**Protein name**	**319_37**	**738_68**	**1248_57**
**Host K-locus**	KL127	KL143	KL28
**K-locus specificity**	KL127	KL143	KL23
**Expression level**	Low	Medium	Medium
**Initial concentration**	320 μg/ml	795 μg/ml	144 μg/ml
**MHFC**	320 μg/ml	0,087 μg/ml	6,125 μg/ml
**Depolymerase activity (spot test)**	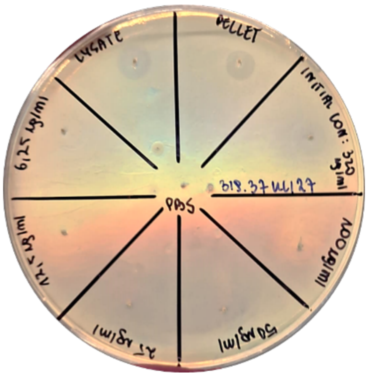	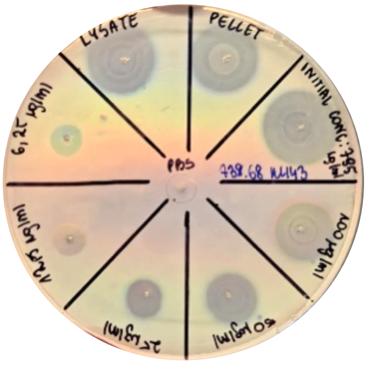	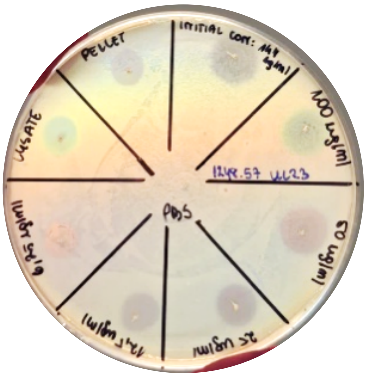

Table 1. Experimental results for all active recombinant proteins identified through manual screening (continued).

Protein name	1251_37	1409_59	1441_47
**Host K-locus**	KL28	KL28	KL28
**K-locus specificity**	KL28	KL23	KL23
**Expression level**	Low	Low	Low
**Initial concentration**	377 μg/ml	130 μg/ml	160 μg/ml
**MHFC**	377 μg/ml	25 μg/ml	1,56 μg/ml
**Depolymerase activity (spot test)**	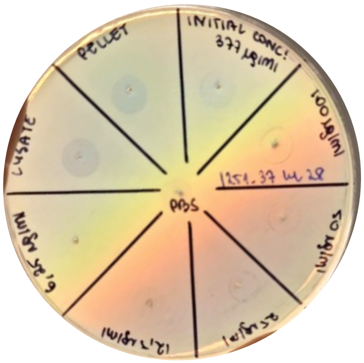	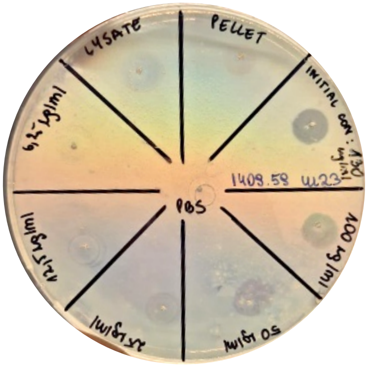	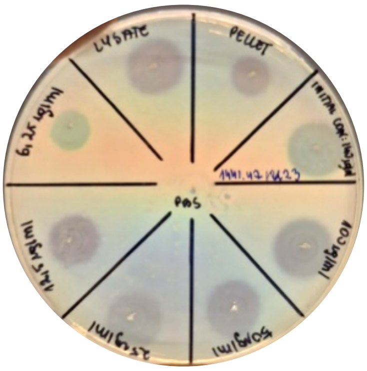

Table 1. Experimental results for all active recombinant proteins identified through manual screening (continued).

Protein name	617_77	434_33	248_38
**Host K-locus**	KL38	KL52	KL55
**K-locus specificity**	KL38	KL52	KL46
**Expression level**	Medium	Medium	Low
**Initial concentration**	434 μg/ml	570 μg/ml	260 μg/ml
**MHFC**	12,5 μg/ml	12,5 μg/ml	6,25 μg/ml
**Depolymerase activity (spot test)**	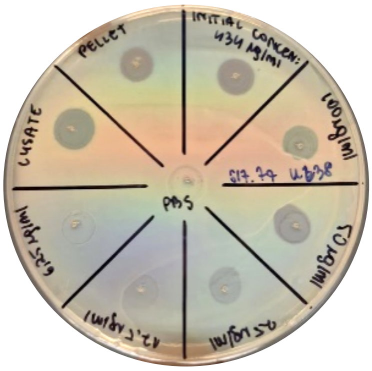	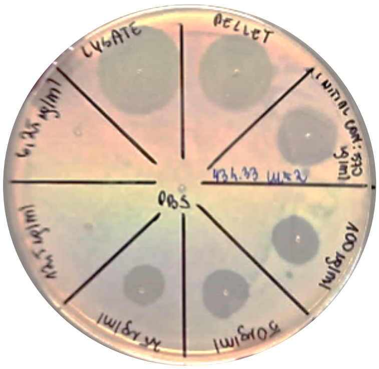	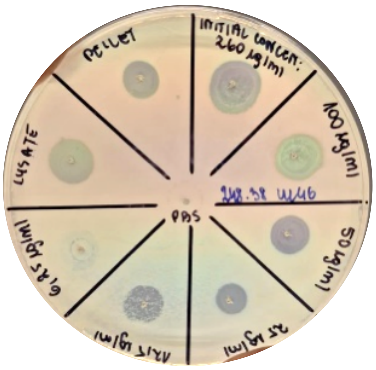

Table 1. Experimental results for all active recombinant proteins identified through manual screening (continued).

**Protein name**	**1723_59**	**1724_71**	**914_74**
**Host K-locus**	KL60	KL60	KL62
**K-locus specificity**	KL60	KL60	KL62
**Expression level**	Low	Low	Low
**Initial concentration**	261 μg/ml	213 μg/ml	120 μg/ml
**MHFC**	261 μg/ml	12,5 μg/ml	3,125 μg/ml
**Depolymerase activity (spot test)**	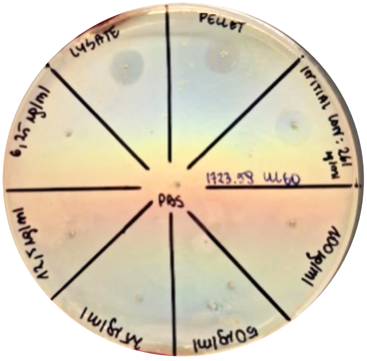	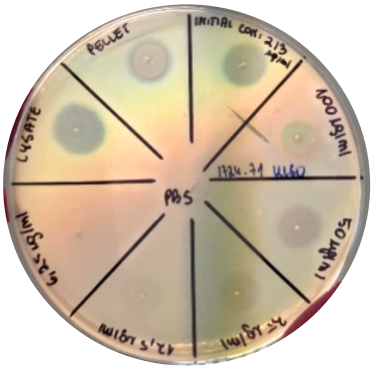	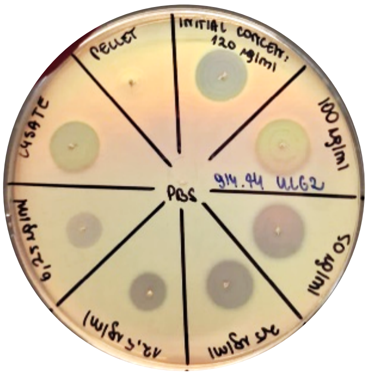

Table 1. Experimental results for all active recombinant proteins identified through manual screening (continued).

**Protein name**	**914_77**	**1091_44**
**Host K-locus**	KL62	KL64
**K-locus specificity**	KL32	KL64
**Expression level**	Low	Low
**Initial concentration**	143 μg/ml	527 μg/ml
**MHFC**	---	25 μg/ml
**Depolymerase activity (spot test)**	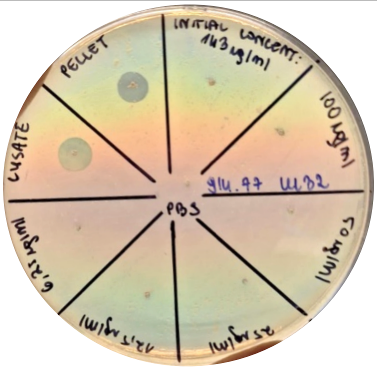	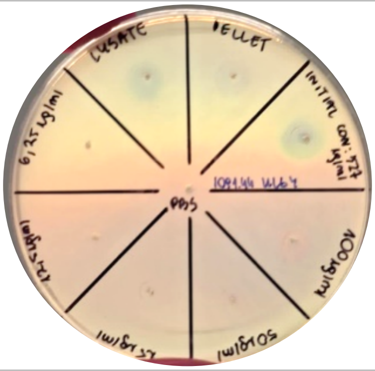

Table 2. Experimental results for all active recombinant proteins identified through GWAS approach. For each protein, the predicted host K-locus (based on prophage origin), experimentally confirmed K-locus specificity, expression level, and minimal halo-forming concentration (MHFC) are shown. Spot tests were performed using purified proteins (tested at the initial concentration and in a 2-fold dilution series). The lowest concentration at which visible capsule degradation (halo formation) occurred was recorded as MHFC.

**Protein name**	**0367_12**	**0574_17**	**0391_11**
**Host K-locus**	KL62	KL14	KL62
**K-locus specificity**	KL62	KL14	KL62
**Expression level**	Medium	Medium	High
**Initial concentration**	360 µg/ml	4,5 mg/ml	1,06 mg/ml
**MHFC**	3,125 µg/ml	6.25 µg/ml	25 µg/ml
**Depolymerase activity (spot test)**	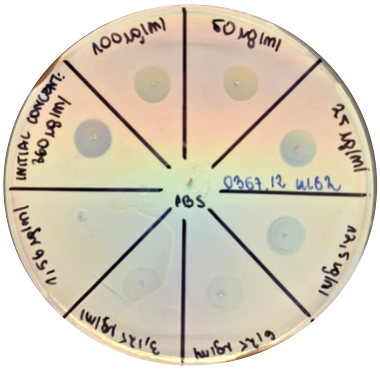	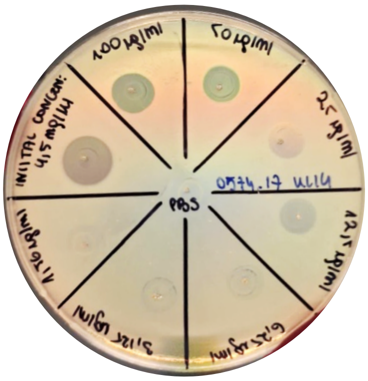	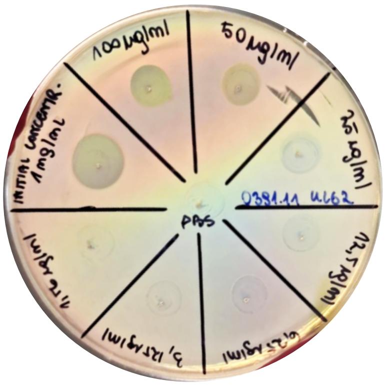

Table 2. Experimental results for all active recombinant proteins identified through GWAS approach (continued).

Protein name	0496_72	391_03	184_43
**Host K-locus**	KL122	KL3	KL111
**K-locus specificity**	KL122	KL3	KL111
**Expression level**	Medium	High	High
**Initial concentration**	710 µg/ml	3,15 mg/ml	5 mg/ml
**MHFC**	1,56 µg/ml	1,56 µg/ml	12,5 µg/ml
**Depolymerase activity (spot test)**	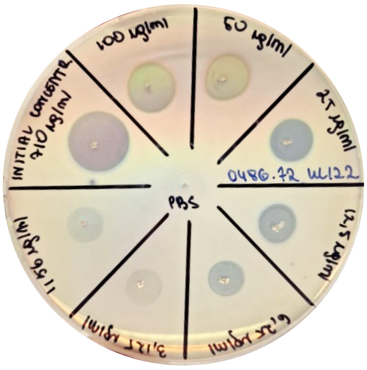	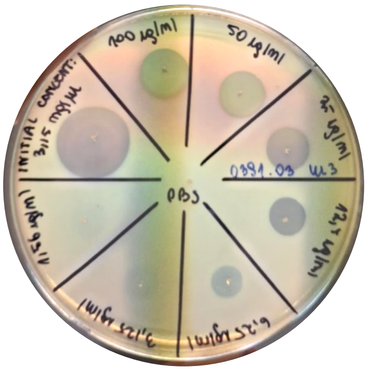	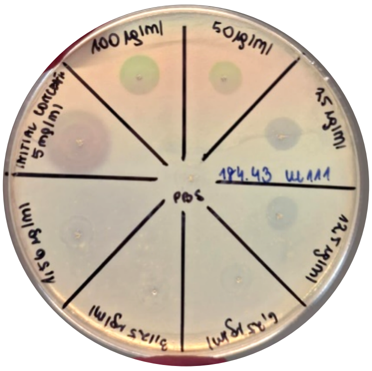

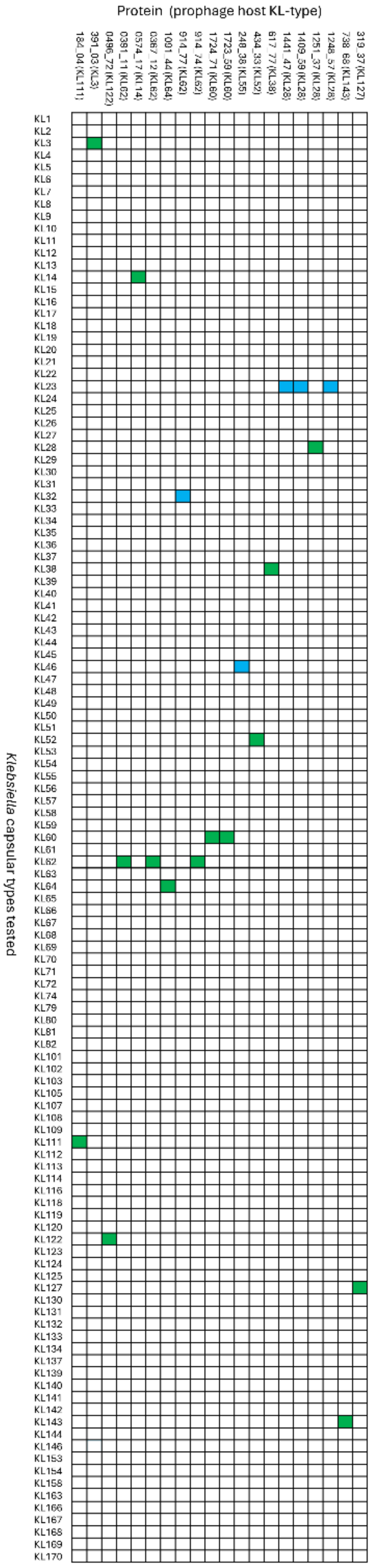

Protein (prophage host KL-type)
184_04 (KL111)
391_03 (KL3)
0496_72 (KL122)
0391_11 (KL62)
0574_17 (KL14)
0367_12 (KL62)
1091_44 (KL64)
914_77 (KL62)
914_74 (KL62)
1724_71 (KL60)
1723_59 (KL60)
248_38 (KL55)
434_33 (KL52)
617_77 (KL38)
1441_47 (KL28)
1409_59 (KL28)
1251_37 (KL28)
1248_57 (KL28)
738_68 (KL143)
319_37 (KL127)
Klebsiella capsular types tested
KL1
KL2
KL3
KL4
KL5
KL6
KL7
KL8
KL9
KL10
KL11
KL12
KL13
KL14
KL15
KL16
KL17
KL18
KL19
KL20
KL21
KL22
KL23
KL24
KL25
KL26
KL27
KL28
KL29
KL30
KL31
KL32
KL33
KL34
KL35
KL36
KL37
KL38
KL39
KL40
KL41
KL42
KL43
KL44
KL45
KL46
KL47
KL48
KL49
KL50
KL51
KL52
KL53
KL54
KL55
KL56
KL57
KL58
KL59
KL60
KL61
KL62
KL63
KL64
KL65
KL66
KL67
KL68
KL69
KL70
KL71
KL72
KL74
KL79
KL80
KL81
KL82
KL101
KL102
KL103
KL105
KL107
KL108
KL109
KL111
KL112
KL113
KL114
KL116
KL118
KL119
KL120
KL122
KL123
KL124
KL125
KL127
KL130
KL131
KL132
KL133
KL134
KL137
KL139
KL140
KL141
KL142
KL143
KL144
KL146
KL153
KL154
KL158
KL163
KL166
KL167
KL168
KL169
KL170

Figure 1. Heatmap of depolymerase activity against Klebsiella pneumoniae capsular types. Each recombinant depolymerase is shown along the X-axis, accompanied by the KL-type of its prophage host. The Y-axis lists all tested *Klebsiella* KL-types. Coloured squares represent positive activity signals, with green indicating activity against the prophage host KL-type and blue indicating activity against a distinct KL-type.
