# Supplementary material for: Capsular specificity in temperate phages of Klebsiella pneumoniae is driven by diverse receptor-binding enzymes
Source: PLoS Biol. 2026 Apr 28;24(4):e3003716. doi: 10.1371/journal.pbio.3003716 (PMC13123978; doi:10.1371/journal.pbio.3003716)
Supplement: Attachment — Submitted filename: PLOS_Biology_response2reviewers.pdf [file pbio.3003716.s029.pdf]

Reviewer #1:

The study by Owtinowska and colleagues uses a genome-wide association approach with experimental validation to identify prophage-encoded proteins with specificity for and activity against Klebsiella pneumoniae capsular loci. The authors identify prophage loci and protein clusters that are significantly associated with specific loci. They also find that protein clusters that are strong predictors of capsule (K) type have a relatively high correlation with functional depolymerase activity against that capsule type. They also explore the types of prophage proteins associated with capsule types and find that SGNH hydrolases are found frequently, and appear to be found in a greater diversity of K types, suggesting they may be important mediators of phage infection. The technical merit of the study is high, with a large number of genomes used from two different collections, and rigorous genomics, statistical, and experimental approaches used. The experimental design is logical
and rigorous, including the synthesis and testing of a large number of prophage proteins. As written, the novelty and significance of the findings is less clear.

Major Comments

Based on the introduction, it is somewhat unclear what the significance of the study is and what the important gap in knowledge is that this study will fill. Was the goal to identify novel proteins that may target the capsule for degradation, perhaps as eventual therapeutics or bioremediation to control Klebsiella populations? Is the focus on integrated prophages a way to efficiently screen for these proteins, as opposed to physical searches for lytic phages in the environment? The gap in knowledge needs to be more explicitly stated.

We have now added a new paragraph in the introduction which explicitly states the three major knowledge gaps that our study aims to address (page 4, lines 88-99). Briefly, these are:

- **<u>Genetic determinants</u>: Which prophage genes control capsule (K-locus) tropism, and do they correspond to the canonical depolymerases known from virulent phages?**
- **<u>Apparent rarity</u>: Why are depolymerase genes so uncommon in prophages — does this reflect a different infection strategy or limits of current functional annotation?**
- **<u>Functional activity</u>: Are prophage-encoded depolymerases active against the capsule of their own host (or other K-types), given that capsule switching enables phage escape?**

Correspondingly, the significance of the results needs to be conveyed relative to the gap in knowledge. It seems that the takeaway is that there was some success in predicting and

validating capsule depolymerases, but that often the proteins in prophages are inactive. Does the identification of these new depolymerases drive the field forward? In addition, there are other categories of phage proteins that may be associated with capsule type and infectivity. These SGNH hydrolases and their potential deacetylase activity is discussed over several paragraphs in the discussion, but these proteins are not experimentally tested and make up a small portion of the results. Overall, I think the discussion needs to convey the broad importance of the findings.

In the revised MS we have rewritten the first part of the discussion to better align our findings with the three knowledge gaps explicitly stated in the introduction (pages 17-18, lines 423-479). Furthermore, we have now experimentally validated two tail fibres with the SGNH hydrolase domain as esterases and added a new Figure 7. We think that these changes have substantially improved the clarity of the MS with respect to the significance of the results.

Minor Comments:

Figure 2A: This is shaded by species but not sequence cluster, contradicting the figure legend.

We have now corrected the Figure legend.

Figure 2B: It would be more helpful to the reader if this showed only the K types included in the analysis, whereas Figure S2 can show all the K types considered.

We have now changed Figure 2B accordingly.

Page 9, lines 16-20: I would suggest moving a brief explanation of precision and recall, F1 and Matthews here, and how they all use TP, FP, TN and FN values for their calculation. This context is important for interpreting the results and figures.

We have added a new fragment in the results which intuitively explains all of these measures (pages 7-8, lines 171-183).

Reviewer #2:

This manuscript presents a comprehensive investigation into the genetic determinants of capsule tropism in temperate phages of K. pneumoniae. The authors analyzed 3,900 Klebsiella genomes (spanning diverse ecological niches) and applied GWAS to correlate prophage protein clusters (from 8,105 prophages) with bacterial K-loci. Furthermore, a total of 60 putative depolymerases were expressed as recombinant proteins, and enzymatic activity was tested on a reference panel of 119 K-types.

The results showed that most predicted depolymerases (34/60) failed to yield soluble products, and 6 lacked activity against the tested strains. Besides, 5 of 14 enzymes displayed activity

against K-types different from those of the prophage host. The authors further proposed that SGNH-domain-containing RBPs might rely on capsule acetylation and that deacetylation could represent a strategy used by temperate phages to recognize and initiate infection.

While the work is ambitious and technically demanding, many conclusions are insufficiently supported by experimental evidence. In particular, most predicted depolymerases failed to yield soluble or active proteins, and proposed roles of SGNH hydrolases in capsule deacetylation remain speculative without functional validation. Overall, the manuscript reports substantial effort but provides limited novel biological insights, raising concerns about its suitability for PLOS Biology.

Major Comments

1. Generality of Study Population: The study population was derived only from Australia (clinical isolates) and Italy (diverse sources). The authors should discuss the implications of this limited sampling for the global diversity of Klebsiella prophages and RBPs.

We thank the reviewer for raising this important point. We fully agree that sampling can influence how generalisable conclusions are to the global *Klebsiella pneumoniae* population. At the same time, we note that no existing dataset is truly globally representative, as most large genomic collections are strongly biased toward clinical isolates enriched for antimicrobial resistance or hypervirulence, and often geographically skewed. Our study aimed to minimise these epidemiological biases by using two systematically collected isolate sets (Australia and Italy) that were not selected based on resistance or virulence traits. We acknowledge, however, that restricting the dataset to two countries does limit the geographic breadth of the sampled diversity.

To evaluate how this might affect the generality of our findings, we performed a new analysis (see a new S15 Figure) comparing all sequence types (STs) in our GWAS dataset with those in two of the largest available global resources:

- **ARMnet, comprising >45,000 publicly available *K. pneumoniae* genomes with standardised MLST and K-locus assignments from the Pathogenwatch online database, and**
- **KlebNNSsero, a recent collection of ~2,000 neonatal bloodstream isolates from 35 sites in 13 countries across Africa and South Asia, assembled without selection for resistance or virulence and therefore representing a broad clinical background.**

Across these comparisons, we found that a substantial proportion of the sequence types in our dataset are globally distributed: 390 of 542 STs (72%) have been isolated in at least two countries, and 347 STs (64%) occur on at least two continents, including all major pandemic lineages such as ST11, ST15 and ST307. The remaining STs appear geographically restricted to Australia or Italy. Thus, although our dataset is

geographically limited, it nonetheless captures a large fraction of the globally circulating *K. pneumoniae* lineages represented in the largest available collections.

We now explicitly discuss these implications in the revised Discussion and include a new supplementary figure summarising the comparison of ST distributions across datasets.

2. Validation of SGNH Hydrolase Function: The proposed O-deacetylation activity of SGNH domain proteins is not experimentally demonstrated. Functional assays are needed to support this claim.

We thank the reviewer for this important comment. In the revised manuscript, we now include functional evidence showing that two prophage-encoded SGNH-domain RBPs display clear esterase activity consistent with O-deacetylating enzymes.

We selected two RBPs with strong GWAS support predicted as containing SGNH domains (164_08 predicted against KL2, and 174_38 predicted against KL55), along with a β-helix depolymerase lacking an SGNH domain (184_43 against KL111) as a negative control. All three proteins were produced as highly purified preparations. To assess enzymatic activity, we used a p-nitrophenyl acetate (pNPA) hydrolysis assay—a widely established test for SGNH hydrolases and carbohydrate esterases, including acetylesterases that remove O-acetyl groups from complex polysaccharides.

Both 164_08-KL2 and 174-KL55 showed robust, time-dependent hydrolysis of pNPA, reflected by a marked increase in absorbance at 405 nm and the expected release of p-nitrophenol. A complementary phenol-red assay confirmed acetate formation through a clear pH-dependent colour shift. In all cases, the SGNH-domain RBPs produced strong, reproducible signals characteristic of active acetyl esterases. These results demonstrate that prophage-encoded SGNH-domain RBPs possess bona fide esterase activity, consistent with the proposed role in polysaccharide deacetylation.

In the revised manuscript, we have added these new data as a dedicated Results subsection (pages 15-16), with a new Figure 7, and updated the Methods accordingly.

3. Protein Expression and Activity: It is premature to conclude that non-expressed proteins are non-functional (p. 24, lines 26-28), particularly as the expression system used failed to yield soluble products for the majority of candidates. Expression vectors, tags, or conditions may affect protein folding and activity. The authors should consider optimizing recombinant expression strategies.

We thank the reviewer for this insightful comment and fully agree that the absence of detectable overexpression does not necessarily imply a lack of functionality; we now state this explicitly in the revised manuscript. Our initial experiments used a single expression system (Champion™ pET101), chosen because minimal tags reduce the risk of disrupting folding or trimerisation of large depolymerases and because this vector has routinely enabled successful expression of *Klebsiella* depolymerases in our laboratory.

The conditions chosen by us are consistent with those reported in the literature for other *Klebsiella* depolymerases (see the new S5C Table). A uniform approach also ensured comparability across the full panel of 50 recombinant proteins from the manual search

In direct response to the reviewer's concern, we undertook a systematic re-expression campaign for eight representative candidates spanning different clusters and predicted structural folds (see S5B Table). We varied induction temperature, inducer concentration and expression duration, including low-temperature induction (15–18 °C) commonly used for large trimeric depolymerases and consistent with both the literature and the conditions used successfully by our external provider. Despite these optimisations, seven of the eight candidates showed no detectable overexpression by SDS–PAGE. One protein expressed at low levels and could be purified, but it remained inactive against all tested *Klebsiella* serotypes. Importantly, activity was assessed not only on purified protein but also directly on crude lysates—allowing detection even when expression is below gel sensitivity—and empty-vector controls confirmed that no background halo activity was present. The results are summarised in the table below and presented in the new S5B Table.

Protein	706_38	1668_49	1668_52	279_60	1057_41	245_48	1096_14	666_83
Host K-type	KL2	KL2	KL2	KL25	KL25	KL55	KL64	KL116
Expression level	undetectable	undetectable	undetectable	undetectable	undetectable	low	undetectable	undetectable
Enzymatic activity	not active	not active	not active	not active	not active	not active	not active	not active
Predicted protein structure (AF2)	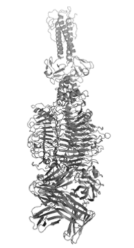	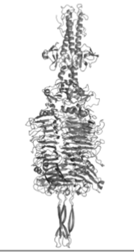	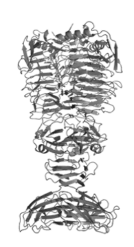	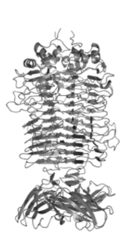	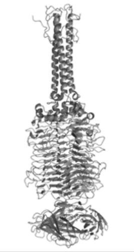	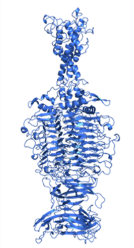	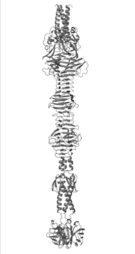	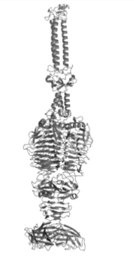

Furthermore, because the initial manuscript hypothesised that non-expression might reflect cryptic or degraded prophages, we performed an additional set of analyses to test this possibility. These results (now shown in new S5-S9 Figures) revealed no evidence that non-expressed candidates originate from degraded or domesticated prophages. Specifically:

- structural predictions for non-expressed proteins showed intact folds with no systematic defects (S5 and S6 Figures);
- prophage genomes non-expressed depolymerases were not more likely to display signs of being cryptic, whether by completeness estimates, transposase content or being domesticated (S7 Figure);
- three high-quality prophages contained both an active depolymerase and a second depolymerase that failed to express (S8 Figure);

- **and, in two striking cases, pairs of proteins sharing >97% amino-acid identity differed dramatically in expression outcome (one active, one non-expressed; S9 Figure).**

Together, these results strongly suggest that the lack of detectable activity for many candidates is not attributable to cryptic prophages or obvious structural degradation but rather reflects the difficulty of heterologously expressing large, multi-domain tail fibres outside their native phage context.

In the revised manuscript, we have extensively modified the Results, Discussion, Methods and included the new supplementary figures and tables mentioned above.

4. Novelty and Impact - Although correlations between prophages and K-loci were examined, most depolymerase predictions remain unvalidated, limiting the study's contribution to understanding capsule specificity.

We thank the reviewer for this thoughtful comment. We understand the concern that many depolymerase predictions cannot be experimentally validated within the scope of this study. We have reflected carefully on this point, and we agree that the original manuscript did not sufficiently foreground the conceptual insights that emerge independently of exhaustive experimental confirmation.

In line with the reply to other reviewers, we wanted to clarify that the principal goal of our work was to uncover the broader genetic and functional logic of capsule recognition in temperate *Klebsiella* phages. In this sense, the experimental screen serves as one component of a much larger integrative framework. The key advances of the study lie in showing that:

- **capsule specificity in temperate phages is mediated by a far more diverse set of receptor-binding proteins than previously recognised,**
- **the perceived scarcity of depolymerases reflects structural and functional diversity, underlying complex co-evolution, rather than biological absence, and**
- **functional activity is difficult to predict from sequence alone, explaining why prophage depolymerases are such an unreliable resource for active enzymes**

We have now ensured that these points are clearly articulated so that the novelty of the study does not appear to hinge on how many enzymatic predictions were validated, but rather on what the combined computational and experimental findings reveal about the fundamentals of phage biology.

At the same time, we have substantially strengthened the experimental component in direct response to the reviewer's request—by validating SGNH-domain RBPs as functional esterases and by re-expressing and retesting expression conditions for recombinant proteins—thereby extending the validation set beyond what is typically feasible for genome-scale analyses.

In the revised manuscript, we clarified the scope of the study by explicitly framing it around the three major knowledge gaps we address (genetic determinants of capsule specificity, the apparent scarcity of depolymerases, and the functional activity of prophage RBPs) and revised the Discussion to interpret our findings in this context. We believe these changes substantially improve the clarity and impact of the work. We are grateful to the reviewer, whose comment prompted revisions that sharpened the presentation of our conceptual advances and helped strengthen the manuscript overall.

Minor Comments

1. Page 10, line 13: Define CATH, PDB, and AFDB at first mention.
2. Page 10, line 24: Remove "and."
3. Page 13, line 28: Correct "brea"
4. Page 13, line 29: remove duplicate "the."

These have now been corrected.

Reviewer #3:

The authors combine large Klebsiella genomic databases to develop a genome wide association study between K-loci and phage proteins. They first mine Klebsiella genomes for prophages, extract these and cluster the proteins, then use these as input for the GWAS, with appropriate controls for the underlying population structure. In parallel they perform functional validation of various candidates from one of the collections. Importantly, they find that very few of the predicted proteins confer the expected functionality.

Overall the analysis is thorough, the manuscript well-written throughout and the results support the conclusions. The choice of datasets is rational and encompasses a range of ecologies. The key result (for me) is that so few of the predicted receptor-binding enzymes appear to be functional in the screening assay. I agree with the authors that this has important consequences for mining prophages for functional enzymes.

I felt there was a slight mismatch between the GWAS analysis and the recombinant expression part of the project. GWAS can identify associations without a reliance on homology (as noted by the authors), but how were the candidate recombinant RBPs found from the prophage genomes? On page 13, line 16 this is simply referred to as 'meeting initial selection criteria' and is presumably homology based? I'd suggest reiterating those criteria here. The authors do a decent of job of aligning the results from these parallel arms of the project in the results, but the introduction could be a bit clearer.

We thank the reviewer for this helpful observation. In the revised manuscript we have now clarified in the Results what exactly we mean by ‘initial selection criteria’.

Specifically, we now state that depolymerase candidates were identified through a manual screen of prophage proteins based on sequence length, functional annotation as tail fibres/spikes, and the presence of predicted polysaccharide-degrading enzymatic domains (lyases or hydrolases), followed by structural modelling with AlphaFold to confirm the presence of a β-helix–type fold. The new fragment can be found on page 10, lines 249-250.

The authors mention degraded / cryptic prophages in the discussion but should consider whether/how these would influence the GWAS? My thinking here is that once non-functional there will be no selection on these genes, which could affect the GWAS. I believe the use of different clustering levels (50%, 80%) would mitigate this effect, but the authors could discuss this.

We thank the reviewer for raising this interesting and important point. To address it, in the revised MS we have rerun the GWAS analyses by restricting the dataset only to prophages marked as "complete" using CheckV. As shown in the new S16 Figure, we found that the obtained results are largely unchanged, measured by the maximum F1-score per K-Locus obtained using all prophage regions vs one obtained using only prophages assigned as 'complete'. A strong correlation between the two metrics was obtained regardless of the clustering threshold. We would also like to point out that the original GWAS results were also obtained for six different clustering thresholds, as now explicitly stated in the Results (pages 9-10, lines 237-238) as we found RBP modularity to strongly impact the obtained GWAS results. We have also included a new paragraph in the Discussion which discusses this issue (page 21, lines 542-554)

Minor points:

Suggest giving an overview of the use of elastic-net regression models and their usage for GWAS. I believe this is an appropriate technique, but an overview would be helpful for non popgen familiar readers.

We have now revised this part of the Results by providing a clearer and intuitive explanation of the reasons for choosing the elastic-net approach (page 7, lines 153-170).

As noted in the discussion, the halo assay is quite limited for inferring activity. Are there other methods that could be used for future work?

The halo effect could have limitations specifically for phage activity testing. The absence of a visible halo during phage activity testing, even when the phage carries depolymerases as virion-associated enzymes, can be explained by virion diffusion limitations in agar. Larger virions tend to diffuse less efficiently through the agar matrix, which reduces the distribution of phages and may prevent the formation of a noticeable halo. In contrast, when testing purified depolymerase enzymes, the halo effect remains one of the most reliable and widely used methods for assessing enzymatic activity and specificity.

However, it is important to note that certain enzymes, such as SGNH-type hydrolases, often cause only partial degradation (e.g., removal of acetyl groups) rather than complete cleavage of capsular polysaccharides (CPS). In such cases, the halo effect will not be observed because CPS remains largely intact.

As mentioned in the discussion, the halo assay has limitations for inferring activity. For future work, alternative approaches could include:

- **Quantitative biochemical assays (e.g., reducing sugar release, HPLC, or colorimetric methods) to measure CPS degradation.**
- **Mass spectrometry or NMR analysis to detect structural changes in polysaccharides.**
- **Viscosity reduction assays for polymer breakdown.**
- **Surface plasmon resonance or ITC for binding and activity characterization.**

These methods provide more precise insights into depolymerase activity beyond the qualitative halo observation, but they require isolation and purification of CPS polysaccharides for precise biochemical/biophysical analyses. Those sophisticated techniques are usually used for single CPS degradation analysis by a specific depolymerase. In our case, we needed to use a higher throughput and a simple method, such as halo formation.

We have now added a short clarification in the Discussion to point to such alternative approaches (page 21, lines 556-560).

Page 2, line 13: 'great model'- slightly colloquial.

We have now corrected this.

Typo, page 26, line 6. 'quasipenumoniae'

We have now corrected this.

Page 26. Line 10- 'all isolates'.

We have now corrected this.

Page 27. Line 19- Code snippets- unfinished sentence. Link to GitHub repo perhaps.

We have now deleted this. All github repos with the relevant code snippets are included in the manuscript ('Reproducibility' section)
