# Supplementary material for: Capsular specificity in temperate phages of Klebsiella pneumoniae is driven by diverse receptor-binding enzymes
Source: PLoS Biol. 2026 Apr 28;24(4):e3003716. doi: 10.1371/journal.pbio.3003716 (PMC13123978; doi:10.1371/journal.pbio.3003716)
Supplement: Attachment — Submitted filename: Response2reviewers.pdf [file pbio.3003716.s030.pdf]

Reviewer #1:

This manuscript by Otwinowska is significantly improved. It is very helpful to lay out the 3 gaps in knowledge in the introduction and return to them in the discussion. This helps frame the article and keep the reader focused on the significant findings. The added functional data on SGNH hydrolase raises the significance of the work, increasing the likelihood that these enzymes enable an alternative infection approach separate from depolymerase activity. The section briefly explaining the metrics of precision and recall is also helpful. I have a few remaining minor comments:

1- Figure 7 photographs do not match the conditions shown in the graphs. This makes their interpretation a bit difficult and raises the concern that some conditions seen in the photograph were not reproducible or were not quantified. It seems like the conditions should match across the graphs and photographs in Figures 7B and C.

We thank the reviewer for pointing this out and making an effort to read and revise our manuscript again. In the revised manuscript, we have aligned the conditions shown in the graphs with those presented in the photographs in Figures 7B and 7C. All quantified conditions are now directly represented in the corresponding images. The only exception concerns the “Protein only” condition: in the graphs, this is shown as the mean OD of the three proteins (as their traces overlapped and were time-independent), whereas the photographs display each protein separately for completeness. This distinction is now clearly explained in the figure legend and in the Methods. In addition, we have provided all raw measurements underlying these panels in S3 Data to ensure full transparency and reproducibility.

2- As a reader, I was still asking myself the question: If we fill the 3 gaps in knowledge, then we can do what? Perhaps it is have a better understanding of the basic biology of temperate phages, and that would be valid but would need some context. But the authors state in the discussion that the findings carry "direct implications for designing more effective phage therapeutics and capsule based vaccines" (Lines 568-570), but I am still not sure how they do that.

We thank the reviewer for highlighting this. In the revised manuscript, we toned down and reframed the translational statements to better reflect the scope of the study to preserve the biological relevance of the work while avoiding overstatement.

Specifically, we revised the final paragraph of the Discussion (page 31, lines 544-549) to avoid implying direct immediate applications. Instead, we now emphasise that our findings suggest capsule structure and modification state may influence phage susceptibility in more complex ways than previously appreciated, and that deeper mechanistic understanding will be important for future work aiming to harness phages therapeutically or design capsule-based interventions.

Furthermore, we clarified that the full implications of acetylation and deacetylation for infection outcomes require future work (page 29, lines 489-491).

Reviewer #2:

In the revised manuscript, the authors have conducted additional experiments, including testing alternative expression conditions for eight representative predicted depolymerases and validating the esterase activity of SGNH-domain RBPs. Additionally, the authors have modified their conclusions/discussion to highlight that capsule specificity in temperate phages involves a diverse set of receptor-binding proteins and that functional activity is difficult to predict from sequence alone.

Overall, the data presented are solid and well-interpreted. The manuscript has been improved by the inclusion of these new results and the refinement of several statements, which is appreciated.

Nevertheless, in my opinion, the current data still do not fully resolve the study's central questions regarding the genetic and functional basis of specificity in temperate phages. Specifically, it remains unclear which prophage genes mediate capsule tropism and what exactly drives capsular specificity in Klebsiella pneumoniae temperate phages. Therefore, despite the revisions, a definitive conclusion regarding the primary driver of specificity is still lacking.

We thank the reviewer for the careful evaluation and for raising important concerns about the genetic and functional basis of capsule specificity in temperate phages. In the revised manuscript, we addressed these points through three coordinated sets of changes.

1. Clarification of terminology (capsular specificity vs. host tropism)

We recognised that confusion may have arisen from inconsistent use of capsular specificity and host tropism. We have now standardised terminology throughout the Abstract, Introduction, Results and Discussion. We use capsule-driven host tropism to describe the phage-level infection phenotype (infection patterns structured by capsule type), and capsular specificity to describe protein-level interactions (e.g., enzyme–capsule specificity assessed computationally and experimentally). In the GWAS section, we also explicitly define genetic predictors of capsular specificity as statistically supported associations between prophage protein clusters and bacterial K-loci, rather than direct measurements of infection outcomes (page 11, lines 155-158).

2. Clarification of objectives and expectations vs. findings

We first clarify the expectations based on virulent (lytic) phages, where capsule-driven host tropism is typically explained by capsule-specific depolymerases encoded in RBPs. We link this established framework explicitly both in the opening of the Abstract and in the Introduction, to clearly separate prior knowledge from the focus of the present study (page 1, line 16; page 3, lines 68-70). We then state more clearly that, for temperate phages, our aim was not to identify a single universal determinant of capsular specificity, but rather to detect recurrent prophage-encoded protein families statistically associated with specific K-loci, and to test representative candidates experimentally (page 4, lines 98-102). Finally, in the Discussion we interpret our results explicitly in the context of these virulent-phage expectations, emphasising that classical β-helix depolymerases remain a major mechanism, while additional enzymatic strategies—particularly SGNH-domain esterases—appear widespread in prophages and may contribute via capsule modification (page 28, lines 436-440).

3. Clarification of conclusions and uncertainty

Finally, we refined the Discussion to better reflect the limits of mechanistic inference. We now emphasise that while GWAS identifies robust predictors and experiments confirm enzymatic activity, further work is required to determine how capsule acetylation state affects adsorption and infection outcomes *in vivo* (page 29, lines 489-491). We also toned down broader implications in both the Abstract and Discussion to avoid overinterpretation (page 2, lines 40-43; page 31, lines 544-549).

Overall, these revisions align terminology, objectives and conclusions more tightly with the data, while preserving the main contribution.

Reviewer #3:

The authors have addressed all of my concerns. I believe the interpretation of results is careful, measured and appropriate. The combination of analysis of a large dataset of systematically collected genomes and high-throughput expression and screening make this paper appropriate for PLoS Biology.

We thank the reviewer for positive feedback and effort to read and revise our manuscript again.
